# Calyx and Corolla as Heliocaminiform Structures Enhance the Reproductive Adaptation of *Brandisia hancei* (Orobanchaceae) to Low Winter Temperatures

**DOI:** 10.1002/ece3.71495

**Published:** 2025-05-22

**Authors:** Yongquan Ren, Ruifeng Sun, Yu He, Xia Jiang, Weilai Chen, Hongyun Xu, Yanyan He, Xiaoling Tian

**Affiliations:** ^1^ College of Eco‐Environmental Engineering Guizhou Minzu University Guiyang China; ^2^ Guizhou Academy of Forestry Guiyang China; ^3^ Guizhou Liping Rocky Desertification Ecosystem Observation and Research Station Qiandongnan China; ^4^ Guiyang College of Humanities and Science Guiyang China

**Keywords:** cold adaptation, floral temperature, heliocaminiform, micrometeorology, reproductive adaptation, thermoregulation

## Abstract

The thermoregulation of plants through the heliocaminiform effect of hollow structures is widespread but overlooked. Winter‐flowering plants in subtropical regions often suffer from low temperatures; however, the reproductive adaptations of these plants are not well understood. The functional advantages of persistent calyx and labiate corolla in *Brandisia hancei* were studied to clarify the mechanism of flower thermoregulation via heliocaminiform structures, and to understand the reproductive adaptation of floral temperature increases in winter. The daily dynamics of floral temperature were recorded under two weather conditions. Floral temperatures were also measured (1) under shaded conditions, (2) after calyx or corolla removal, and (3) after a hole was made or after the hole in the corolla was sealed. Seed production was then evaluated after perianth manipulation. The heliocaminiform effect of persistent calyx and labiate corolla was obvious under sunny conditions, and a maximum temperature excess up to 10°C was recorded. However, the temperature excess disappeared under overcast to rainy conditions or after shading. The removal of either the calyx or corolla generally reduced floral temperatures compared to intact flowers. Making a hole in the corolla also led to a slight decrease in floral temperature, which was partially restored after sealing the hole. Consistent with these thermal effects, the removal of the calyx and corolla even at later stages caused a reduction in seed production. Notably, making only a small hole in the corolla reduced seed production, whereas the number of seeds also rebounded after the hole was sealed. By effectively increasing floral temperature, the heliocaminiform effect of the calyx and corolla enhances the reproductive fitness of *B. hancei* . Our study presents empirical evidence for floral thermoregulation via heliocaminiform structures and for reproductive adaptation through floral temperature increases in winter conditions in subtropical regions.

## Graphical abstract

The empirical evidence for the heliocaminiform effect in flowers is strikingly scarce, and the reproductive fitness of this kind of floral thermoregulation has not been well evaluated. Both the calyx and corolla are beneficial for increasing floral temperature, and the heliocaminiform effect of the calyx and corolla facilitates seed production in *Brandisia hancei* in winter. The results will contribute to understanding the reproductive adaptation mechanism of winter‐flowering plants in subtropical regions.

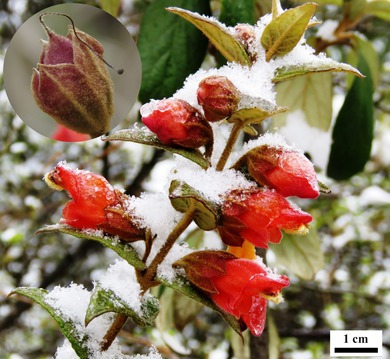

## 1 Introduction

Temperature is an important factor for the sexual reproduction of plants and affects many aspects of reproductive processes, such as gametophyte development (Hedhly 2011; Ohnishi et al. 2010), pollen germination (Distefano et al. 2018; Sherer et al. 2024; Wagner et al. 2017), and seed development (Gao et al. 2019; Rosbakh et al. 2018). Even short‐term low temperatures may affect one or more of these processes and ultimately hinder the successful reproduction of plants (Takeda and Glenn 2016; Wagner et al. 2017). Plants are susceptible to low ambient temperatures in high‐altitude or high‐latitude regions (Kevan 2020; van der Kooi et al. 2019), whereas in midlatitude regions, plants also experience low temperatures in winter. Under low‐temperature conditions, many plants attain temperatures above that of the ambient air by which they improve reproductive fitness (Dietrich and Körner 2014; Zhang et al. 2017). Depending on the species, plants apply various strategies to maintain a suitable floral temperature. Some plants have evolved thermogenesis to increase floral temperature through active metabolic heating (Claudel et al. 2023; Wang et al. 2014), whereas more plants increase floral temperature through passive solar heating mechanisms. These include changeable flower orientation (Atamian et al. 2016; Creux et al. 2021; Koski et al. 2024), darker flower coloration (Lacey et al. 2010; Little et al. 2016), and anatomical adaptations (Kevan and Coates 2024; Song et al. 2024; van der Kooi et al. 2019).

As anatomical adaptations, hollow structures can elevate internal temperatures through a “microgreenhouse effect,” whereby heat is absorbed and retained within the enclosed chamber (Kevan 2020; Kevan and Coates 2024). Enclosed floral structures (e.g., corolla and calyx) function as typical microgreenhouses, regulating temperature and safeguarding reproductive organs from low‐temperature stress, thereby improving reproductive success (Abdusalam and Tan 2014; He et al. 2006; Hou et al. 2022; Liu et al. 2017; Sherer et al. 2024). Open hollow structures formed by some flowers are also assumed to promote heat accumulation and effectively increase floral temperature (Kevan 2020; Kevan and Coates 2024). Some “glasshouse plants” are covered by translucent bracts, which can also effectively help plants adapt to low‐temperature stress via hollow structures. *Rheum nobile* is a typical “glasshouse plant” with translucent bracts covering the inflorescences. Its bracts can effectively achieve flower warming by transmitting solar radiation, thus promoting the development of flowers and enhancing pollination, fertilization, and seed development (Omori et al. 2000; Song et al. 2013, 2020).

Hollow structures in plants are widespread and highly diverse, playing a crucial role in thermal regulation (Kevan 2020; Kevan and Coates 2024). Although terms such as “greenhouse” and “glasshouse” are commonly used to describe transparent or translucent plant structures that facilitate sunlight penetration, they seem to be inadequate for characterizing the broader phenomenon of plant thermoregulation through hollow structures. Tissue translucency is typically classified based on visible light transmittance, yet radiative absorption is inherently wavelength‐dependent (Jacques 2013), varying across solar spectral components—particularly in the infrared range. Notably, many hollow plant structures with limited translucency, including stems and fruits, achieve considerable elevated temperatures in their internal spaces (Kevan 2020; Kevan and Coates 2024). Consequently, the conventional “greenhouse effect” framework inadequately accounts for these thermal strategies in optically opaque tissues. Kevan and Coates (2024) thus introduced the term “heliocaminiform” to describe the form and function of hollow structures in plants, a novel and overlooked field in botany. In our study, we accordingly use the term heliocaminiform to describe the thermoregulatory role of hollow structures in plants. Although the thermal properties of hollow structures created through corolla closure have been relatively well‐studied (e.g., Abdusalam and Tan 2014; Hou et al. 2022; Imamura and Hariu 2020; Liu et al. 2017), empirical research on heliocaminiform structures is strikingly scarce (Kevan 2020; Kevan and Coates 2024). Moreover, the reproductive fitness of this floral thermoregulation mechanism has yet to be thoroughly assessed. Additionally, the vast majority of research on low‐temperature floral thermoregulation has been conducted in alpine or arctic ecosystems, leaving the adaptive strategies of subtropical winter‐flowering plants largely unexplored.

**FIGURE 1:**
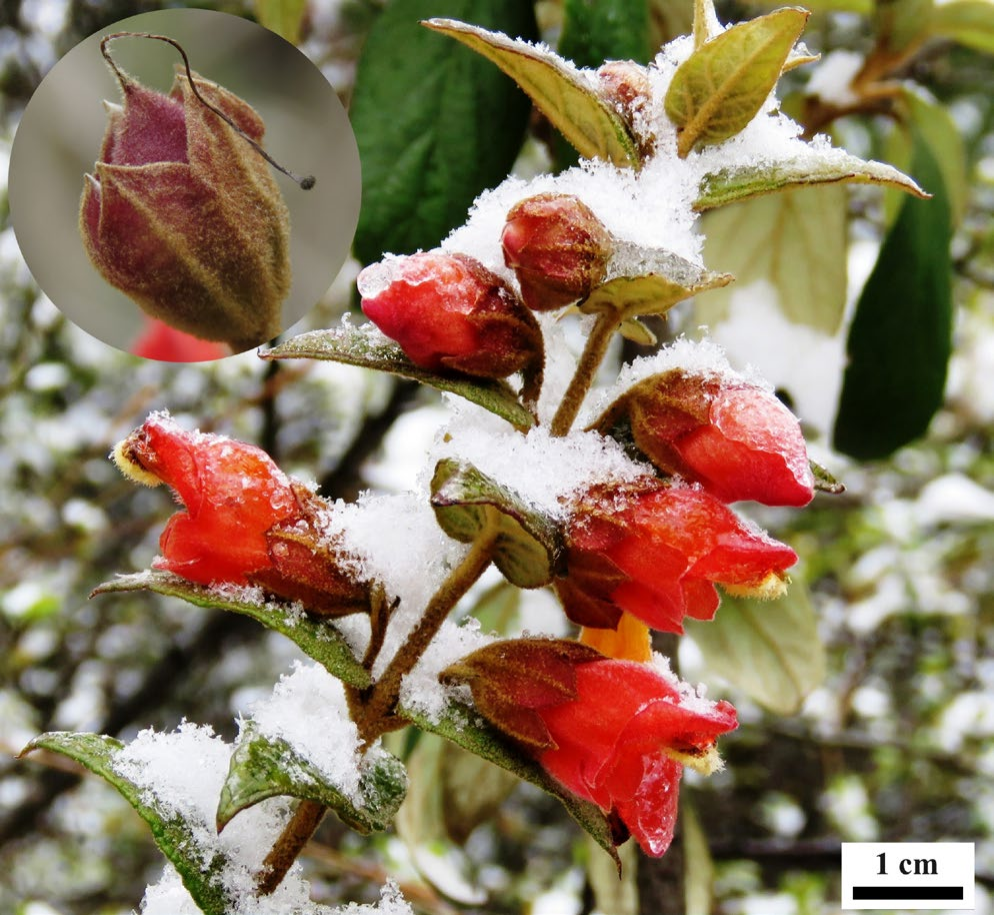
Flowers of *Brandsia hancei* blossoming in winter. The enclosed corolla forms a chamber before anthesis, the labiate corolla forms a hollow space after opening, and the persistent calyx partly encloses the developing fruit (insert).

*Brandisia hancei* Hook. f. is a winter‐flowering shrub that grows in subtropical regions in China (Hong et al. 1998). The flowers of *B. hancei* bear a persistent campanulate calyx and a labiate corolla, and usually suffer from low temperatures in winter (Figure 1). At early floral stages, the enclosed corolla emerges from the bud, forming a chamber before anthesis, and then the corolla opens, forming a hollow space (Figure 1). The calyx persists after anthesis, partly enclosing the developing fruit (Figure 1, insert). *B. hancei* is an ideal model for investigating reproductive adaptation to harsh winter conditions from the perspective of the heliocaminiform effect of its calyx and corolla. The objective of our study was to determine the role of the calyx and corolla in floral thermoregulation and the reproductive adaptation of floral temperature increases in *B. hancei* . We tested the hypotheses that the calyx and corolla functioned as heliocaminiform structures and that an increase in floral temperature enhanced the reproductive fitness of *B. hancei* under winter conditions.

## 2 Materials and Methods

### 2.1 Study Species and Sites

*Brandisia hancei* blooms in winter, from December to March of the following year. This plant has been reported to be pollinated by the passerine bird *Yuhina nigrimenta* (Qian et al. 2017), and delayed self‐pollination also facilitates its reproduction in winter through the self‐contact of anthers with the stigma before withering (Ren et al. 2023). For the convenience of experimental description, three key stages during anthesis were recognized: (1) an enclosed corolla formed a chamber, and the flower was opening (Opening); (2) corolla newly opened (Opened); and (3) corolla had withered and fallen, leaving behind the persistent calyx (Fallen).

Field experiments were carried out in two natural populations of *B. hancei* in Guizhou Province, SW China. Both experimental sites were secondary shrublands: one was located in Daozhen County (29.0337 N, 107.6471 E; 735 m a.s.l.), and the other was in Huishui County (26.1623 N, 106.7634 E; 1245 m a.s.l.). The experimental sites have a subtropical monsoon climate with low winter temperatures. Meteorological data obtained from https://lishi.tianqi.com indicate that January, the coldest month at both sites, frequently experiences subzero temperatures. During our field experiments, the lowest recorded temperature in Daozhen was −3°C (January 2021). Similarly, in Huishui, the extremely low temperature dropped to −5°C in January 2024.

### 2.2 Temperature Measurement for Natural Flowers

In our study, all temperatures were measured in the Huishui population with multichannel thermocouple thermometers (TA612C; Suzhou TASI Electronics Co. Ltd., Suzhou, China). When floral temperature was measured, the probe was inserted into the bottom of the flower, close to the ovary. The probe for measuring ambient temperature was placed behind an adjacent leaf to avoid rain or sunlight.

To test the potential floral temperature increment above the ambient temperature, daily temperatures were recorded on 17th December 2023 and 31st January 2024. The former day was overcast to rainy, and the latter day was cloudy. One flower at the Opening stage and another adjacent flower at the Opened stage were selected. The floral temperatures of these two flowers and the nearby ambient temperature were simultaneously measured at 6‐min intervals for 24 h. Given that the increase in floral temperature was significant only under sunny conditions, a sunshade experiment was conducted to clarify the effect of solar radiation. Two opened flowers from the same plant were selected: one was insolated under sunny conditions, and the other was shaded with a sunshade umbrella. The sunshade with vinyl sunscreen is opaque and blocks light from entering. Floral and nearby ambient temperatures for two flowers were measured simultaneously at 1‐min intervals for 30 min on 27th December 2023, a cloudy day.

### 2.3 Temperature Measurement After Perianth Manipulation

Temperature measurements after perianth manipulation were also conducted in the Huishui population in 2024 and 2025. To clarify the effect of the calyx on the floral temperature of *B. hancei* , calyx removal was conducted for flowers at the three stages of Opening, Opened, and Fallen, respectively. At the Opening and Opened stages, the calyx was completely removed, and the corolla remained. The floral temperatures of a calyx‐removed flower and an adjacent intact flower at the same stage, as well as the ambient temperature, were recorded simultaneously. At the Fallen stage, two adjacent withered flowers without corollas were selected, with one serving as the intact control (CK) and the other subjected to calyx removal. As the corolla had already fallen, distinguishing between ambient temperature and floral temperature after total calyx removal was difficult. We therefore only measured the temperature inside the calyx after partial removal, which meant that approximately one‐third of the upper part of the calyx was cut off while retaining the lower two‐thirds. The temperatures inside the intact calyx and partly removed calyx, and the ambient temperature behind an adjacent leaf were recorded simultaneously.

To study the role of the corolla in the increase in floral temperature, the corolla was removed at the two stages of Opening and Opened. At each stage, the floral temperatures of an intact flower, an adjacent corolla‐removed flower, and the ambient temperature were recorded simultaneously. In addition, three adjacent flowers at the Opening stage were further used for each treatment: (1) making a tiny hole in the corolla with a fine‐tipped tweezer (MH), (2) sealing the hole with Vaseline (SH), and (3) an intact flower as the control (CK). The diameter of the hole was approximately 2 mm, and it was located on the side of the middle of the corolla, outside the edge of the calyx. The temperatures inside the three flowers were measured simultaneously.

For each treatment mentioned above, temperature measurements were conducted using 18 replicates (three in 2024, 15 in 2025) collected from distinct individuals. For each replicate, the temperature was measured at 1‐min intervals for 10 min on 31st January and 1st February 2024, and on 27th and 28th February 2025. The temperature measurements were only conducted under sunlight, avoiding cloud obstruction as much as possible.

### 2.4 Seed Production After Perianth Manipulation

To test the effects of the perianth on the reproductive success of *B. hancei* , the calyx and corolla were removed separately at later stages, with the stigma remaining intact. The calyx was removed at the Fallen stage, after the corolla had dropped off. The corolla was removed between the Opened and Fallen stages, after the anthers contacted the stigma. All perianth‐removal treatments were conducted in January 2021 in Daozhen and were repeated in January 2023 in Huishui. To further study the effects of the corolla on the reproduction of *B. hancei* , two corolla treatments were conducted with opening flowers in January 2022 in Daozhen, and were repeated in February 2025 in Huishui: (1) MH and (2) SH. Intact flowers were used as the control for all the perianth manipulations. Thirty flowers located in the upper part of the branches from different individuals were used for all the treatments. To eliminate the potential decrease in natural pollination caused by perianth manipulation, all flowers at the stage of Opened were hand‐pollinated with pollen grains from other individuals. Fruits were collected at the beginning of April, and a few fruits had been gnawed by herbivorous larvae; therefore, only intact fruits were used to count the number of seeds. Mature seeds of *B. hancei* develop membranous wings that expand their size to 3–4 × 1–2 mm (Ren et al. 2021), allowing direct visual quantification without magnification.

### 2.5 Data Analysis

The temperature increase inside flowers is presented as the temperature excess, the difference between floral temperature and nearby ambient temperature (Kevan 2020). To compare the temperature excess under sunny and shaded conditions, the temperature excesses under the two conditions were calculated respectively, and then analyzed by a paired‐samples *t*‐test.

For calyx removal at three stages and corolla removal at two stages, the temperature excesses for intact controls and perianth‐removed flowers were compared. Temperature excesses for intact flowers at stages of Opening and Opened were also compared. To assess the impact of making a hole on floral thermoregulation, the difference in floral temperature between CK and MH, and that between CK and SH were also calculated and then compared. For all perianth manipulation experiments, 10 temperature excess values per flower were calculated, and then the arithmetic mean of these 10 values was utilized. All temperature comparisons mentioned above were analyzed by a paired‐samples *t*‐test.

A generalized linear model (GLM) with a Poisson distribution and log‐link function was used to assess differences in seed production among three perianth‐removal treatments: calyx removal, corolla removal, and intact control. Seed production under three corolla treatments (MH, SH, and CK) was also analyzed by a GLM. In both models, seed number was modeled as the dependent variable, with treatment and year/site as fixed factors. All analyses were performed using IBM SPSS 26.0.

## 3 Results

### 3.1 Measurement of the Temperature of Natural Flowers

**FIGURE 2:**
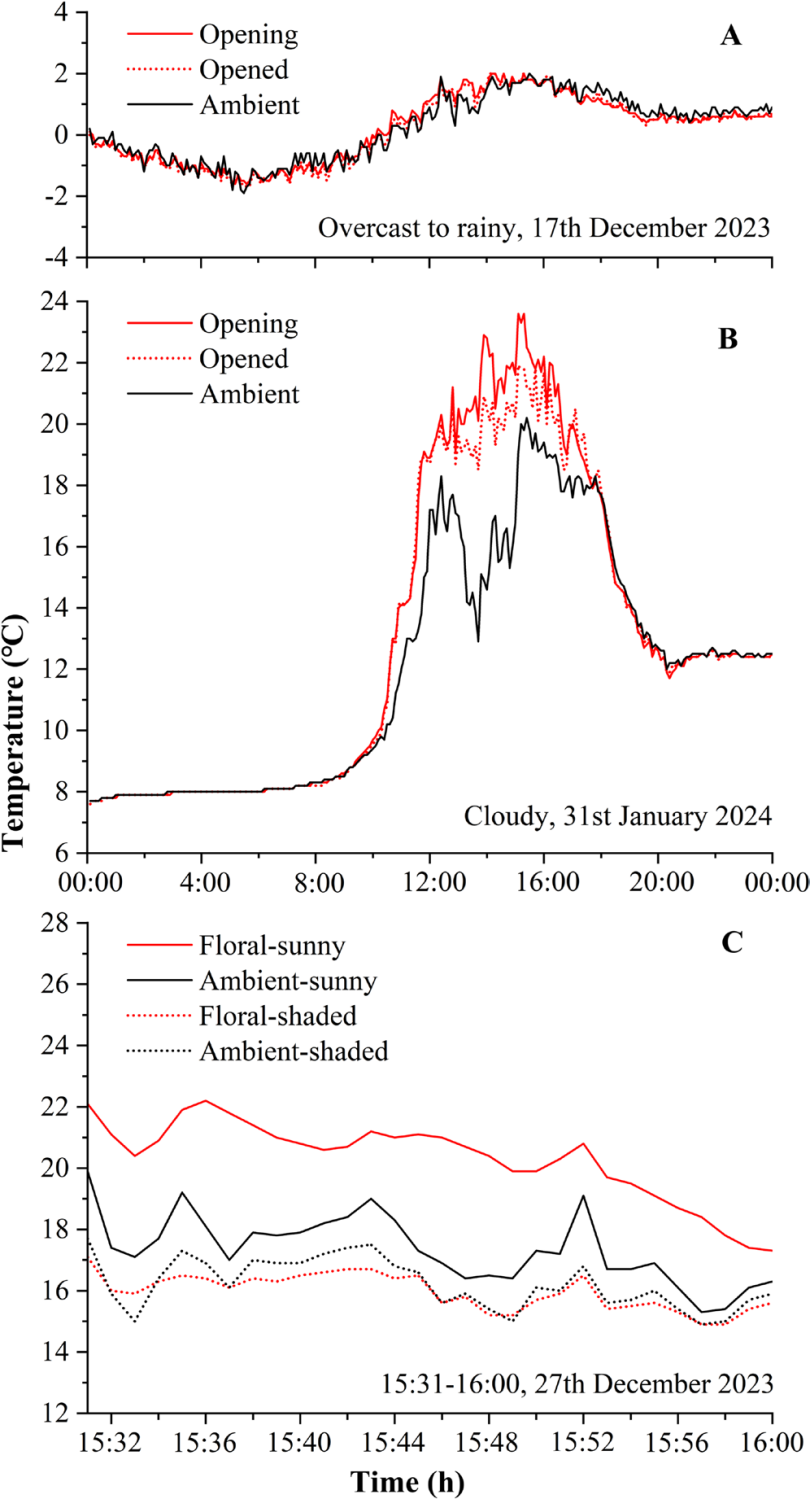
Floral temperatures under different weather and shading conditions. Daily dynamics of floral temperatures were measured at two floral stages on an overcast to rainy day (A) and on a cloudy day (B). Floral and ambient temperatures were also measured under sunny and shaded conditions (C). Opening, the flower is about to open; Opened, corolla newly opened; Ambient, the ambient temperature.

On 17th December 2023, an overcast‐rainy day, floral temperatures fluctuated with the ambient temperature at the Opening and Opened stages, and three temperature curves intertwined, indicating no significant temperature excess throughout the whole day (Figure 2A). On 31st January 2024, a cloudy day, there was also no temperature excess at night for the flowers at the two stages. However, the temperature excesses were obvious during the day, especially when the flowers were exposed to sunlight. Most of the time, the floral temperature of opening flowers was greater than that of opened flowers (Figure 2B). Under both sunny and shaded conditions, floral temperature fluctuated with ambient temperature, and temperatures were always higher under sunny conditions than under shaded conditions (Figure 2C). The paired‐samples *t*‐test revealed a statistically significant difference in the temperature excess between sunny and shaded conditions (*t* = 21.582, df = 29, *p* < 0.001). On average, the temperature excess was 3.0°C under sunshine but disappeared under shade, and the ambient temperature was 0.3°C higher than the floral temperature after shading (Figure 2C).

### 3.2 Temperature Measurements After Perianth Manipulation

**FIGURE 3:**
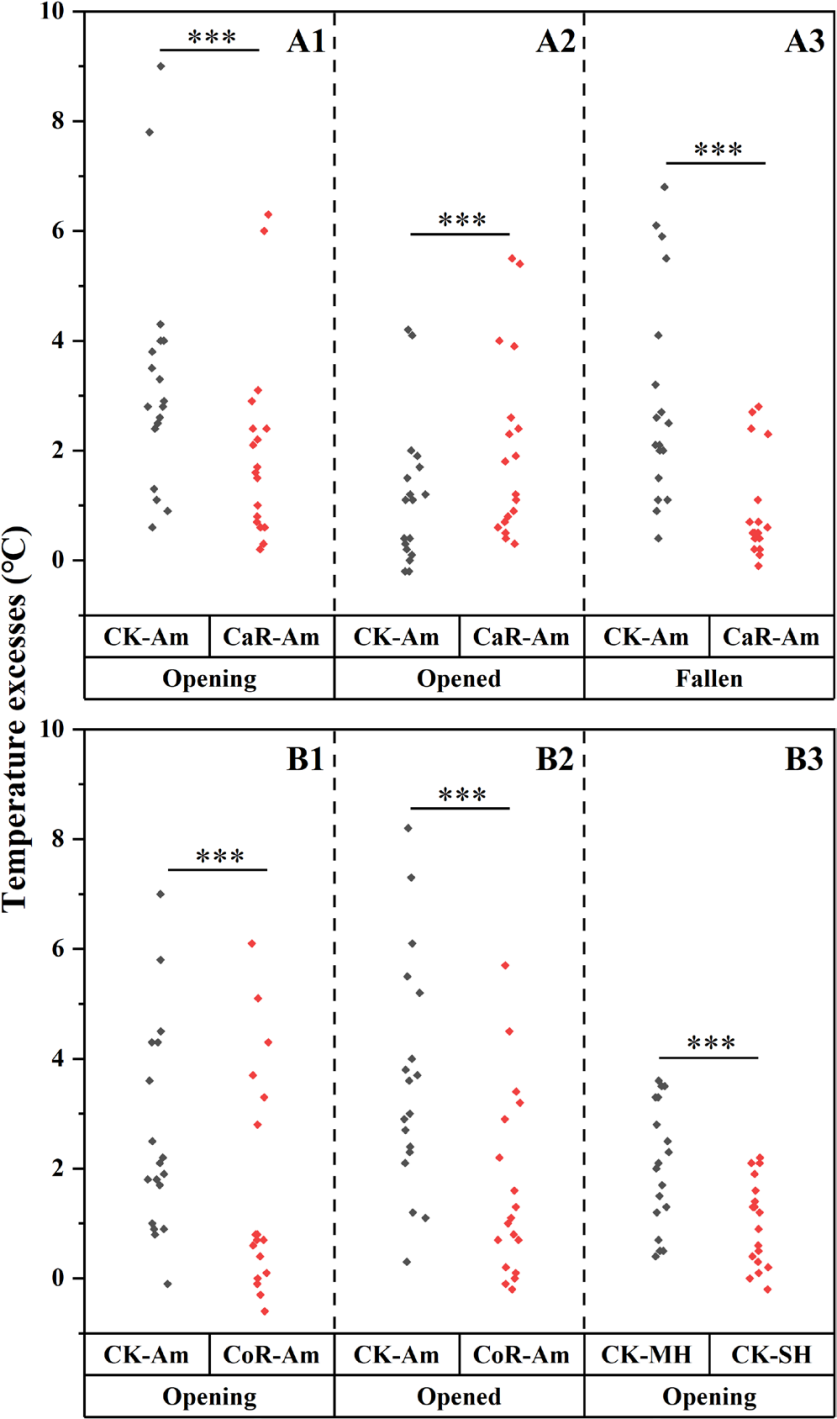
Temperature excesses of flowers following perianth manipulation at different stages. After calyx removal, temperatures were measured when flowers were about to open (Opening, A1), corolla newly opened (Opened, A2), and corolla had withered and fallen (Fallen, A3). For corolla manipulation, temperatures were measured when flowers were about to open (Opening, B1), corolla newly opened (Opened, B2), and after making a hole (MH) or sealing the hole (SH) in the corolla of flowers about to open (Opening, B3). Intact flowers were used as the controls (CK), which temperature excess was calculated as the difference between the temperature inside an intact flower and that of ambient air (CK‐Am). The temperature excess after calyx removal (CaR) was defined as the difference between the floral temperature and the ambient air temperature (CaR‐Am). The temperature excess after corolla removal (CoR) was defined as the difference between the floral temperature and the ambient air temperature (CoR‐Am). After making a hole and sealing the hole, the differences in floral temperatures between intact control and MH (CK‐MH), and that between intact control and SH (CK‐SH) were also compared. ***p < 0.001.

Flowers at the opening stage consistently maintained a higher temperature relative to ambient air, even after calyx removal (Figure 3A1). The average temperature excess was 3.3°C for 18 intact opening flowers, which significantly decreased to 2.0°C after calyx removal (*t* = 6.913, df = 17, *p* < 0.001). For opened flowers, floral temperatures were also always higher than the ambient temperature; unlike opening flowers, calyx removal in opened flowers resulted in an increased floral temperature compared with that of intact flowers (Figure 3A2). An average temperature excess of 1.2°C was recorded for intact opened flowers, which increased to 2.0°C after calyx removal (*t* = −4.917, df = 17, *p* < 0.001). After the corolla fell, the temperatures inside the calyx were also consistently greater than the ambient temperature (Figure 3A3). An average temperature excess of 2.9°C was recorded, and the value significantly decreased to 0.9°C after partial calyx removal (*t* = 7.036, df = 17, *p* < 0.001).

For flowers at the Opening stage, floral temperatures were also always higher than the ambient temperature even after corolla removal, although floral temperature generally decreased slightly after corolla removal (Figure 3B1). The average temperature excess for intact opening flowers was 2.6°C, and the value significantly decreased to 1.6°C after corolla removal (*t* = 7.138, df = 17, *p* < 0.001). The floral temperature of the opened flowers was also greater than the ambient temperature regardless of manipulation, and corolla removal generally resulted in a slight decrease in floral temperature (Figure 3B2). An average temperature excess of 3.6°C was recorded for intact opened flowers, which significantly decreased to 1.6°C after corolla removal (*t* = 5.384, df = 17, *p* < 0.001). Making and sealing holes affected floral temperatures (*t* = 8.135, df = 17, *p* < 0.001). Compared with the intact controls, the average floral temperature decreased by 2°C after making the holes, but the decrease in floral temperature narrowed to 1°C after sealing the holes (Figure 3B3).

Although opening flowers exhibited greater mean temperature excess than opened flowers (3.0°C vs. 2.4°C), this difference was not statistically significant (*t* = 1.607, df = 35, *p* = 0.117). The peak temperature excess values for both developmental stages were observed simultaneously at 14:38 on 27 February 2025, with a flower at the Opening stage reaching 10.0°C, compared to 9.7°C in a fully opened flower.

### 3.3 Seed Production After Perianth Manipulation

**FIGURE 4:**
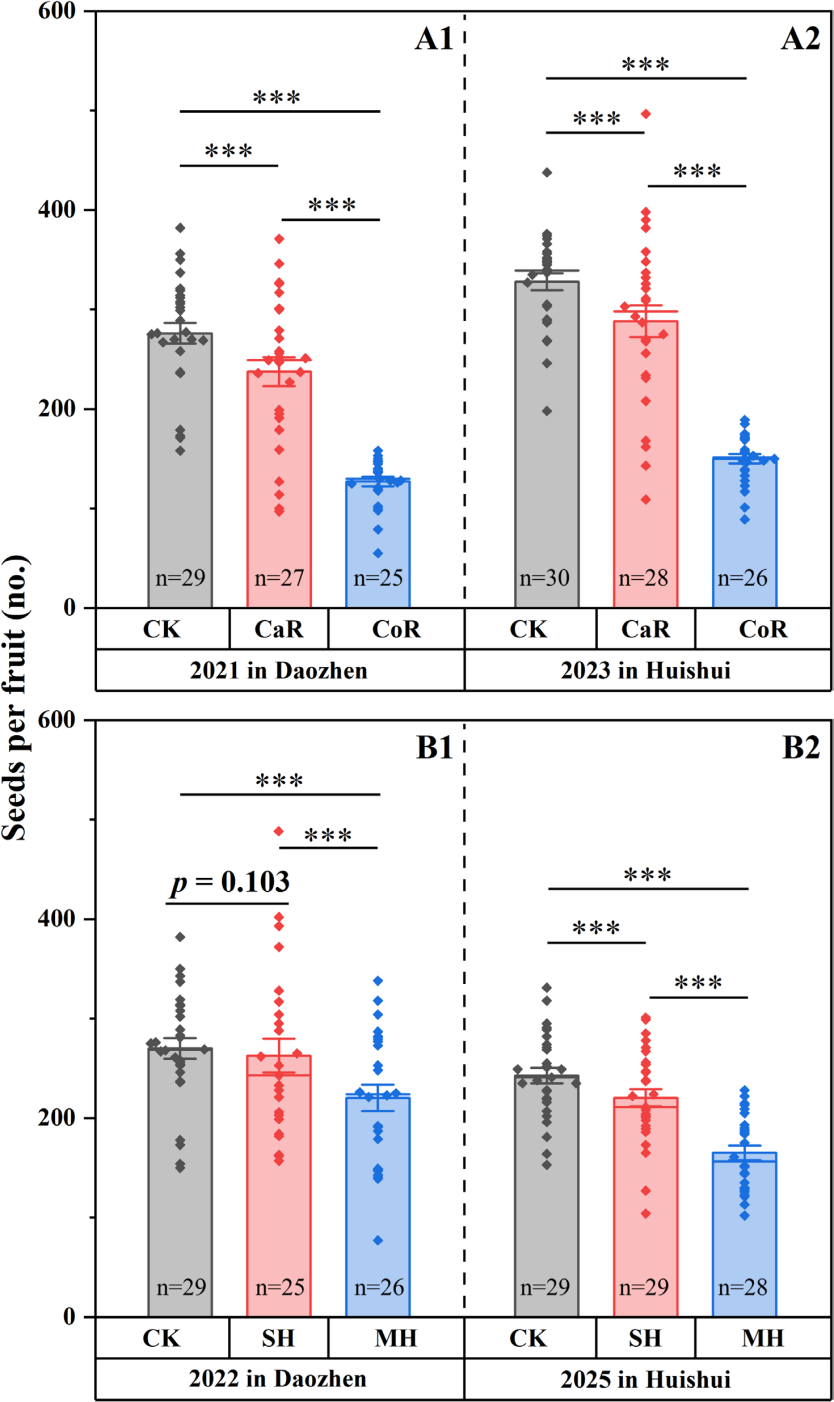
Seed production after perianth removal and corolla manipulation. Experiments for the calyx and corolla removal were conducted in Daozhen in 2021 (A1) and repeated in Huishui in 2023 (A2), with treatments of calyx removal (CaR), Corolla removal (CoR), and intact control (CK). For corolla manipulation, three treatments were set up: making a hole in the corolla (MH), sealing the hole with Vaseline (SH), and intact control (CK). Corolla manipulation was conducted in Daozhen in 2022 (B1) and repeated in Huishui in 2025 (B2). ***p < 0.001. Error bars represent ±1 SE.

The GLM analysis revealed that perianth removal significantly reduced seed production relative to intact controls (Wald *χ* ^*2*^ = 3102.866, df = 2, *p* < 0.001). All pairwise comparisons among treatments (calyx removal, corolla removal, and intact control) showed statistically significant differences (*p* < 0.001) in seed production in both 2021 (Figure 4A1) and 2023 (Figure 4A2). Notably, corolla removal consistently reduced seed production compared to calyx removal in both 2021 (Figure 4A1) and 2023 (Figure 4A2).

The GLM analysis also revealed that making and sealing holes in the corolla had significant effects on seed production (Wald *χ* ^*2*^ = 553.318, df = 2, *p* < 0.001). Specifically, making a small hole led to a significant reduction in seed production in both 2022 and 2025 (both *p* < 0.001). Notably, sealing the hole restored seed production to levels comparable to the control in 2022 in Daozhen (*p* = 0.103, Figure 4B1) and resulted in a significant rebound in 2025 in Huishui (*p* < 0.001, Figure 4B2).

## 4 Discussion

### 4.1 The Mechanism of Floral Temperature Increase

In our study, floral temperatures fluctuated with ambient temperatures, showing evident temperature excesses but disappearing when shading. This suggests that thermogenesis is unlikely to be the mechanism involved, as thermogenesis typically enables plants to maintain relatively stable floral temperatures (Dieringer et al. 2014; Wang et al. 2014). Furthermore, thermogenic plants usually develop large floral structures, either as flowers (Dieringer et al. 2014; Wang et al. 2014) or inflorescences (Bian et al. 2021; Claudel et al. 2023). This morphological adaptation reduces the surface‐to‐volume ratio, minimizing heat loss (van der Kooi et al. 2019). The flowers of *B. hancei* are approximately 2 cm long and 1 cm wide, and are much smaller than thermogenic flowers, such as those of *Nelumbo lutea* and *Magnolia sprengeri* (Dieringer et al. 2014; Wang et al. 2014). Taking the factors mentioned above into account, a reasonable explanation for the temperature excess in the flowers of *B. hancei* is passive solar heating rather than active floral thermogenesis.

The campanulate calyx forms an open hollow structure that initially partially encloses the corolla and later persists to partially encompass the maturing fruit in *B. hancei* (Figure 1). Similarly, the labiate corolla forms a closed space at the early stage, followed by an open hollow structure after blooming (Figure 1). The calyx and corolla of *B. hancei* represent classic hollow structures, creating heliocaminiform structures that facilitate floral thermoregulation (Kevan and Coates 2024). Our experiments demonstrated their critical role in winter thermal adaptation: removal of either calyx or corolla reduced floral temperatures in most cases, with even one tiny hole in the corolla causing temperature reduction (Figure 3). These findings collectively indicate that both calyx and corolla function as efficient heliocaminiform structures, significantly contributing to heat accumulation and retention in *B. hancei* .

Although lacking quantitative measurements, the corolla seems more translucent than the calyx by visual observation, readily allowing solar radiation transmission into the flower. This optical property facilitates passive solar heating, a critical mechanism for floral thermal accumulation (Zhang and Tang 2023). Our study showed that calyx removal led to elevated temperatures in opened flowers compared to intact controls (Figure 3A2). This thermal elevation likely results from the enhanced sunlight penetration through the translucent corolla in the absence of calyx obstruction. Compared to intact flowers, floral temperatures decreased even after sealing the hole in the corolla based on measurements in 2 years (Figure 3B3). Although sealing the hole restored seed production to the level of intact controls in 2022 (Figure 4B1), the 2025 SH treatment showed a significant decline (Figure 4B2). Temperature measurements were conducted using a total of 18 replicates, in which 15 were completed in 2025. After sealing the hole, the floral temperature in 15 flowers still decreased by 1.2°C in 2025, whereas SH treatment restored floral temperatures to the level of CK in 2024. Compared to previous experiments, we used larger Vaseline patches for sealing holes in 2025. This modification may have partially obstructed light, inadvertently altering floral temperatures and subsequently reducing seed production.

Heliocaminiform structures enhance internal warming through three principal thermal mechanisms: radiation, conduction, and convection (Kevan and Coates 2024). In *B. hancei* , solar radiation is absorbed by floral tissues, where it is converted into thermal energy either at the surface or after transmission into the flower. Concurrently, thermal conductivity facilitates heat transfer from the heated surface to the floral interior. The corolla initially forms an enclosed space, and the calyx and corolla subsequently form open hollow structures, limiting convective heat loss under cold conditions. Further contributing to thermoregulation, the calyx and corolla exhibit dense pubescence (Figure 1), a known adaptive trait in alpine plants (Peng et al. 2015; Yang et al. 2008; Yang and Sun 2009). Pubescence thickens the boundary layer and likely suppresses airflow, thereby minimizing convective heat loss (van der Kooi et al. 2019). Collectively, the heliocaminiform structure formed by the calyx and corolla in *B. hancei* integrates these radiative, conductive, and convective optimizations to achieve efficient thermal accumulation and retention.

Following natural corolla abscission, and after the artificial removal of corolla at the Opening and Opened stages, the calyx served as the sole heliocaminiform structure. Corolla also functioned as the sole heliocaminiform structure after calyx removal. Both the calyx and corolla function effectively as heliocaminiform structures for heat accumulation (Figure 3). However, their thermal contributions could not be directly compared in our study, as simultaneous measurements were not performed on flowers at the same stage following separate removal of the corolla and calyx. During anthesis, both calyx and corolla function as heliocaminiform structures, whereas during the fruiting period, the calyx functions independently. The calyx and corolla function as heliocaminiform structures throughout both the flowering and fruiting periods, helping *B. hancei* adapt better to harsh winter conditions.

### 4.2 Floral Temperature and Its Impact at Different Floral Stages

Although opening flowers exhibited a slightly greater mean temperature excess than opened flowers, this observed trend did not attain statistical significance. There is a strong positive correlation between floral temperature increase and flower size (Dietrich and Körner 2014; Zhang et al. 2017; Zhang and Tang 2023). At the early floral stage, the enclosed corolla forms a chamber, but the internal space is limited; flowers at later stages have relatively larger volumes inside the corolla, but the open structure is not conducive to preserving heat (Figure 1). Floral temperature dynamics depend on the balance between heat gain and loss. When the heat accumulation benefiting from the large space inside the flower exceeds the heat loss caused by the open structure, the increased volume leads to higher internal temperatures. Therefore, in some cases, the temperature inside opened flowers may also be higher than that of opening flowers (Figure 2B).

The average temperature excesses decreased after calyx removal at the stage of Fallen (Figure 3A3), and after corolla removal before natural withering (Figure 3B1,B2). Consistent with findings in *Physalis floridana* , where a small decrease in temperature (< 2°C) could have a tremendous effect on reproductive success (Li et al. 2019), our study demonstrated that both calyx and corolla removal significantly reduced seed production compared to intact flowers. Notably, corolla removal exerted a more pronounced negative effect than calyx removal (Figure 4A1,A2). Compared with the control, corolla removal before withering caused only a short‐term decrease in floral temperature, which may have affected pollen germination and fertilization. The calyx was removed when the corolla had dropped off and the fruit had started to grow; thus, a decrease in temperature after calyx removal was more likely to affect seed development. Our previous studies revealed that cold waves may affect pollen development (Ren et al. 2023) and that low temperatures (< 18°C) hinder pollen germination in *B. hancei* (Ren and Sun). Although many developmental stages are susceptible to low temperature (Distefano et al. 2018; Gao et al. 2019; Ohnishi et al. 2010; Wagner et al. 2017), reproductive success seems to be more susceptible at early stages (Li et al. 2022; Rosbakh et al. 2018). Therefore, earlier corolla removal resulted in less seed production than later calyx removal did in our study.

### 4.3 Potential Damage Caused by Experimental Manipulation

Removing either the calyx or corolla, and making a hole in the corolla, all result in decreased seed production compared with that of the intact controls (Figure 4). To minimize potential effects on seed production, corollas were removed only at the later floral stage (proximate to natural withering), and the calyx was removed only after anthesis. Although the removal of plant organs is a commonly employed technique for functional verification studies (Li et al. 2019, 2022; Song et al. 2013), it is still undeniable that the removal of the calyx and corolla is destructive, and thus may cause damage to reproductive success in *B. hancei* . For such experiments, the evidence will be more credible if an additional control is set up: cutting along the edge of floral structures as small as possible to simulate the mechanical damage from experimental manipulations while preserving organ functionality (Gao et al. 2019).

To reduce the potential effect of experimental manipulation on reproductive success, only a small hole was made in the corolla. The floral temperature decreased after making a hole, but the decrease in floral temperature narrowed after sealing the hole (Figure 3B3). Short‐term low temperatures may affect reproductive processes (Takeda and Glenn 2016; Wagner et al. 2017), and a slight temperature increase may benefit reproductive success (Distefano et al. 2018; Ida and Totland 2014). Although seed production significantly decreased in the MH treatment, the SH treatment showed considerable recovery (Figure 4B). Notably, in the 2022 Daozhen trials, we observed no statistically significant difference in seed yield between treatments of CK and SH (Figure 4B1). The synchronous changes in floral temperature and seed production highlight the functional significance of increased floral temperature.

In *B. hancei* , the stigma protrudes from the perianth during anthesis and maintains this exposed position. Thus, both the stigma and any deposited pollen grains are unlikely to be covered and protected by either the corolla or calyx. The perianth manipulation may alter the spatial arrangement of reproductive organs, potentially decreasing contact duration between the anthers and stigma. This mechanical disruption could reduce self‐pollination opportunities, thereby affecting the reproductive success (Ren et al. 2023). To eliminate the potential decrease in pollination opportunities caused by perianth manipulation, all flowers were hand‐pollinated in our study. When potential influences including structural protection and pollination opportunity are excluded, the observed reduction in seed production can be reasonably attributed to the reduction in temperature after corolla and calyx removal.

## 5 Conclusions

The reproductive adaptation of *B. hancei* was studied by examining the roles of the calyx and corolla in maintaining floral temperature and assessing the consequent effects on seed production in a low‐temperature environment. Both the calyx and corolla function as heliocaminiform structures, elevating floral temperature to enhance seed production in winter. Our findings provide experimental evidence for heliocaminiform structures, advancing knowledge in this neglected botanical field, and enhancing our understanding of the reproductive adaptations of winter‐flowering plants in subtropical regions.

## Author Contributions

**Yongquan Ren:** conceptualization (lead), funding acquisition (lead), investigation (lead), methodology (equal), writing – original draft (lead), writing – review and editing (lead). **Ruifeng Sun:** investigation (equal), methodology (equal). **Yu He:** investigation (equal). **Xia Jiang:** investigation (supporting), resources (equal). **Weilai Chen:** investigation (supporting). **Hongyun Xu:** writing – review and editing (equal). **Yanyan He:** writing – review and editing (equal). **Xiaoling Tian:** writing – review and editing (equal).

## Conflicts of Interest

The authors declare no conflicts of interest.

## Data Availability

The data analyzed or presented in this study are openly available at Dryad (https://doi.org/10.5061/dryad.15dv41p54).
